# A review of policies and coverage of seasonal influenza vaccination programs in the WHO Eastern Mediterranean Region

**DOI:** 10.1111/irv.13126

**Published:** 2023-03-21

**Authors:** Rania Attia, Abdinasir Abubakar, Joseph Bresee, Osama Mere, Wasiq Khan

**Affiliations:** ^1^ WHO Regional Office for the Eastern Mediterranean Cairo Egypt; ^2^ The Task Force for Global Health Decatur Georgia USA

**Keywords:** EMR, influenza, policy, survey, vaccination, WHO

## Abstract

**Background:**

Although there has been an effective seasonal influenza vaccine available for more than 60 years, influenza continues to circulate and cause illness. The Eastern Mediterranean Region (EMR) is very diverse in health systems capacities, capabilities, and efficiencies, which affect the performance of services, especially vaccination, including seasonal influenza vaccination.

**Aims:**

The aim of this study is to provide a comprehensive overview on country‐specific influenza vaccination policies, vaccine delivery, and coverage in EMR.

**Materials and Methods:**

We have analyzed data from a regional seasonal influenza survey conducted in 2022, Joint Reporting Form (JRF), and verified their validity by the focal points. We also compared our results with those of the regional seasonal influenza survey conducted in 2016.

**Results:**

Fourteen countries (64%) had reported having a national seasonal influenza vaccine policy. About (44%) countries recommended influenza vaccine for all SAGE recommended target groups. Up to 69% of countries reported that COVID‐19 had an impact on influenza vaccine supply in the country, with most of them (82%) reporting increases in procurement due to COVID‐19.

**Discussion:**

The situation of seasonal influenza vaccination in EMR is varied, with some countries having well established programs while others having no policy or program; these variances may be due to resources inequity, political, and socioeconomic dissimilarities. Few countries have reported wide vaccination coverage over time with no clear trend of improvement.

**Conclusion:**

We suggest supporting countries to develop a roadmap for influenza vaccine uptake and utilization, assessment of barriers, and burden of influenza, including measuring the economic burden to enhance vaccine acceptance.

## 1 INTRODUCTION

Seasonal influenza is a contagious acute respiratory infection caused by influenza viruses, which circulate in all parts of the world.^1^, ^2^ Up to a billion people get seasonal influenza every year with the increasing fear of influenza pandemic; hence, there is an urgent need to monitor circulating respiratory viruses, including influenza.^3^ This monitoring informs the vaccine composition recommendations that WHO issues twice a year.^4^ Influenza causes high morbidity and mortality around the world, contributing to more than 4.6 million cases from 149 countries globally with 3 million cases of severe disease and half a million deaths annually.^5^ Vaccination is considered the most effective control measure against influenza in spite of the presence of number of antiviral drugs approved by Food and Drug Administration (FDA) and heir availability in Eastern Mediterranean Region (EMR).^6^ The protection acquired from these vaccines is limited to the vaccine antigen and fades by time.^7^ Despite this, annual vaccination is highly recommended to all age groups, and the strain used in these vaccines is based on the updated data in the surveillance report of World Health Organization (WHO) laboratories.^8^ Influenza vaccination is one of the key components of the strategic objectives of the global influenza strategy (2019–2030),^9^ which advocates expansion of seasonal influenza prevention and control policies and programs to protect vulnerable.^10^ The WHO strategic advisory group of experts on immunization (SAGE), which was established by the Director‐General of the WHO in 1999 to provide guidance on the vaccines, has recommended seasonal influenza vaccination for high‐risk groups as a high priority such as older adults (over 65 years), health workers, pregnant women, and individuals with underlying health conditions.^11^ To reduce overall burden of influenza, including morbidity and mortality, CDC recommends annual flu vaccination as long as flu activity is ongoing once yearly as a first effective protective strategy against influenza infections.^12^ Although the availability of safe, effective, and well‐tolerated influenza vaccines with rare significant side effects have been in use for more than 60 years, the uptake of influenza vaccines in EMR remains sub‐optimal.^13^ There are several challenges, such as lack of local evidence, competing health priorities, limited collaboration among stakeholders, and vaccine hesitancy and misconceptions.^14^ Consequently, in EMR, influenza vaccination implementation and use vary among countries.^15^

Seasonal influenza epidemics can lead to severe economic drops through loss of workforce productivity, whether by increased morbidity, mortality, or overwhelming the capacity of health services.^16^ That is why it is necessary to estimate BoD to revive influenza vaccination and convince decision‐makers to strengthen their programs.^17^

We would like to provide an overview of influenza vaccination in EMR to better understand gaps and work needed to strengthen influenza vaccination uptake and utilization in the region. We will describe country‐specific influenza vaccination policies, distribution and delivery, coverage, COVID‐19 vaccine deployment, influenza vaccination, and public awareness of influenza vaccination in EMR.

## 2 METHODS

An analysis of (1) a retrospective data from the WHO‐UNICEF‐Joint Reporting Form (JRF) for the period of 2017–2021 was conducted (these data are collected through a standard questionnaire sent to all countries^18^); (2) data from the 2021–2022 influenza season regional survey of EMR; and (3) data and information generated regularly by the countries.

Because reporting was inconsistent in the JRF, therefore, to compliment the findings, a regional survey was conducted to collect more robust and comprehensive data. These data were collected using the regional survey on seasonal influenza vaccination policies and programs (2021–2022). A similar survey was conducted previously (2016–2017), and this regional survey (2021–2022) was developed using a standardized self‐administered questionnaire, used in 2016–2017, composed of 41 questions for collecting data on vaccine policy, programs, distribution points, coverage, and target groups. Survey link was sent to the influenza vaccination focal persons and/or EPI managers at the Ministries of Health via emails, and the responses were compiled in early September 2022. Consistency and level of data completeness varied for different variables in both the JRF and regional survey, and because of this incompleteness issues, we were unable to undertake analyses of all variables from each country.

Variables covered by both the JRF and EMR survey were vaccination policies, target groups, licensure, national immunization program plans and policies, AEFI, roadmap to increase influenza vaccination use, awareness, procurement, COVID‐19 vaccine deployment, delivery point, delivery method, cost, type of vaccine, formulation, vaccine availability, doses distributed, vaccination coverage, population coverage, BoD assessment, economic assessment, barrier assessments, and influenza hospitalizations and deaths.

Data collected through the survey was cleaned and validated by WHO at both country and regional levels. Any discrepancies or missing data were excluded, and analysis was done by Influenza team of EMRO.

## 3 RESULTS

Nineteen out of 22 member states (all except Djibouti, Libya and Yemen) responded to the survey with a response rate of 86.4%; we compiled those with 18 EMR countries responded to JRF in 2021. Below, we provide results of different variables which we studied.

### 3.1 Broader findings

#### 3.1.1 National influenza policies and recommendations

Fourteen (64%) out of the 22 EMR countries had reported having a national seasonal influenza vaccination policy. Of them, seven (UAE, Iran, Iraq, Oman, Saudi Arabia, Morocco, and Kuwait) reported that they had updated their national influenza vaccination policy recently within the last 5 years. The main reasons were due to funding changes, inclusion, or emphasis of additional priority groups, increasing of vaccine coverage and change to type of influenza vaccine used.

Jordan and Syria have no national policy but have recommendations targeting certain risk groups with influenza vaccine. Six (27%) countries reported having no policy or recommendations (Afghanistan, Djibouti, Pakistan, Sudan, Somalia, and Yemen).

#### 3.1.2 Implementation of SAGE recommendations

**FIGURE 1:**
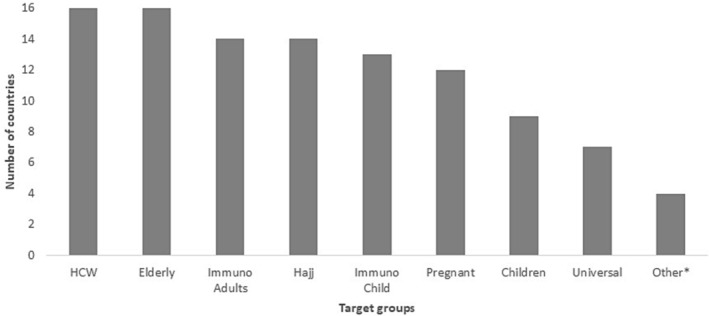
Distribution of SAGE recommended vaccination target groups included in member states' policies/recommendations in the WHO Eastern Mediterranean Region, 2022.

Combining the member states with national policy or recommendations, 7 out of 16 (44%) countries (Bahrain, Iraq, Kuwait, Lebanon, Qatar, Saudi Arabia, and UAE) recommended influenza vaccine for all SAGE recommended groups. Even though Lebanon did not report having universal recommendations for influenza vaccination, they recommended vaccine for all SAGE groups. Egypt, on the other hand, reported having universal influenza vaccination recommendations; however, pregnant women were not included. (Figure 1).

#### 3.1.3 Vaccine regulation and type

Fourteen (14/22; 64%) countries used their national regulatory agencies to approve influenza vaccine use with majority using both southern and northern formulations (9/16; 56%) and the others using only northern hemisphere formulation. Trivalent inactivated vaccine (TIV) is the most used influenza vaccine in the region (15/16;94%); quadrivalent inactivated vaccine (QIV) use has been increasing. There was only one other reported vaccine used by Saudi Arabia, which was a split variant vaccine type.

#### 3.1.4 Vaccine delivery

Influenza vaccine is primarily delivered through Primary Health Care centers and hospitals (12/16; 75%) and outpatient clinics (7/16; 43.8%). The main delivery strategy is through fixed sites (14/16; 88%); however, vaccines were also delivered by eight mobile units and on two locations they used drive through system.

Thirteen (81%) countries reported providing influenza vaccine free to either certain groups or universally. Bahrain reported providing influenza vaccine free of charge only for Bahraini, and vaccine costs were covered for those who were insured. Iran reported covering of costs for prisoners and nursing care centers, in addition to their other target groups.

#### 3.1.5 Vaccination coverage and data

Ten (10/16; 63%) countries reported routinely collecting vaccination coverage data for their targeted groups. Six of the same countries reported that influenza vaccination coverage is collected as part of routine immunization. Only six (38%) countries reported that they have data, which have both numerator and denominator of the covered population.

Of the 16 countries that reported having influenza vaccination policies or recommendation to all SAGE target groups, less than 50% reported having target coverage and (<44%; 7/16) reported actual coverage in the three preceding influenza seasons (Table 1).

**TABLE 1:** The target coverage and reported coverage for the 2018–2019, 2019–2020, and 2020–2021 season.

Target group	Coverage (2018–2019)	Coverage (2019–2020)	Coverage (2020–2021)	Target (2021–2022)
Pregnant women	5.7–95% Reported by 3/16	10.5–96% Reported by 4/16	8.75–92% Reported by 4/16	95–100% Reported by 4/16
HCW	38–82% Reported by 4/16	45.3–81% Reported by 4/16	67.5–84% Reported by 4/16	60–100% Reported by 8/16
Children	1.9–8.3% Reported by 2/16	11.8% Reported by 1/16	3.14–11.5% Reported by 2/16	No % reported Reported by 1/16
Immunocompromised	2.5–86.8% Reported by 3/16	1.6–88.3% Reported by 4/16	8.3–87% Reported by 4/16	40–100% Reported by 5/16
Older adults	2–90% Reported by 3/16	2.9–93% Reported 2/16	15.3–95% Reported by 3/16	90–100% Reported by 3/16
Hajj	100%Reported by 2/16	No % reported Reported by 1/16	Hajj canceled for this season	100% Reported by 5/16
Comments	86.8% only reported for renal patients in Saudi Arabia	88.3% only reported for renal patients in Saudi Arabia	87% only reported for renal patients in Saudi Arabia	100% only reported for renal patients in Saudi Arabia

Challenges in collection and subsequent calculations of vaccination coverage were reported by many countries (13/16; 81%). Beyond the most reported challenge of lack of denominator data, other challenges included difficulties in coordinating with different providers and a shortage of vaccine supply to cover all target groups in the country.

#### 3.1.6 Influenza vaccination program

Five countries (Bahrain, Iran, Kuwait, Saudi Arabia, and Qatar) reported inclusion of influenza vaccine in their National Immunization Programme (NIP), and Oman reported having a plan to do so. Majority of the countries (14/16; 88%) reported having a plan to introduce or expand seasonal influenza vaccination in the coming 5 years in their countries, with only nine countries (9/16; 56%) reporting to have a roadmap to achieve this goal. In terms of vaccine safety, 10 (63%) countries reported having a reporting system for adverse events following immunization (AEFI) of influenza vaccine; eight of those countries also reported their influenza vaccine AEFI system was part of their routine immunization.

**FIGURE 2:**
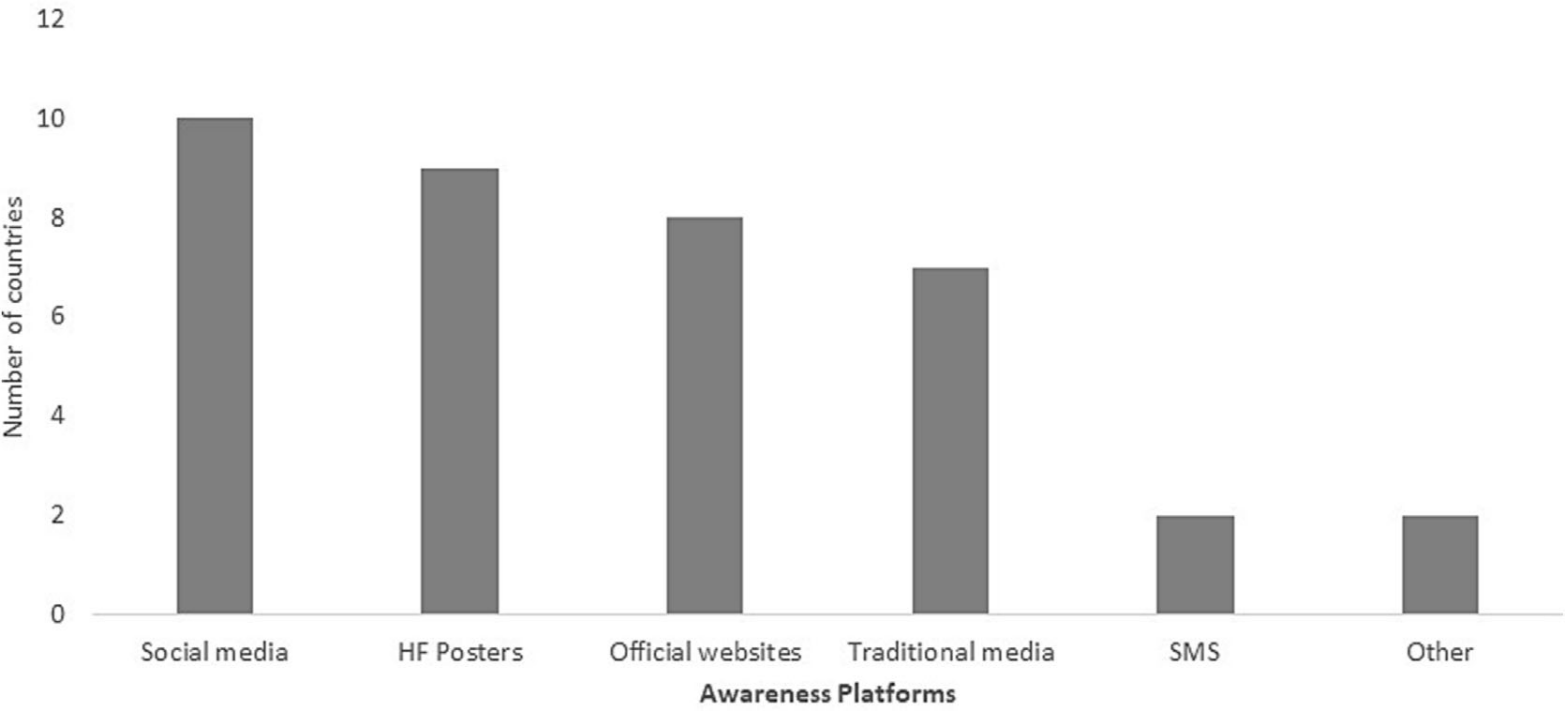
Awareness raising platforms used to promote the uptake and use of influenza vaccine in the WHO Eastern Mediterranean Region, 2022.

**FIGURE 3:**
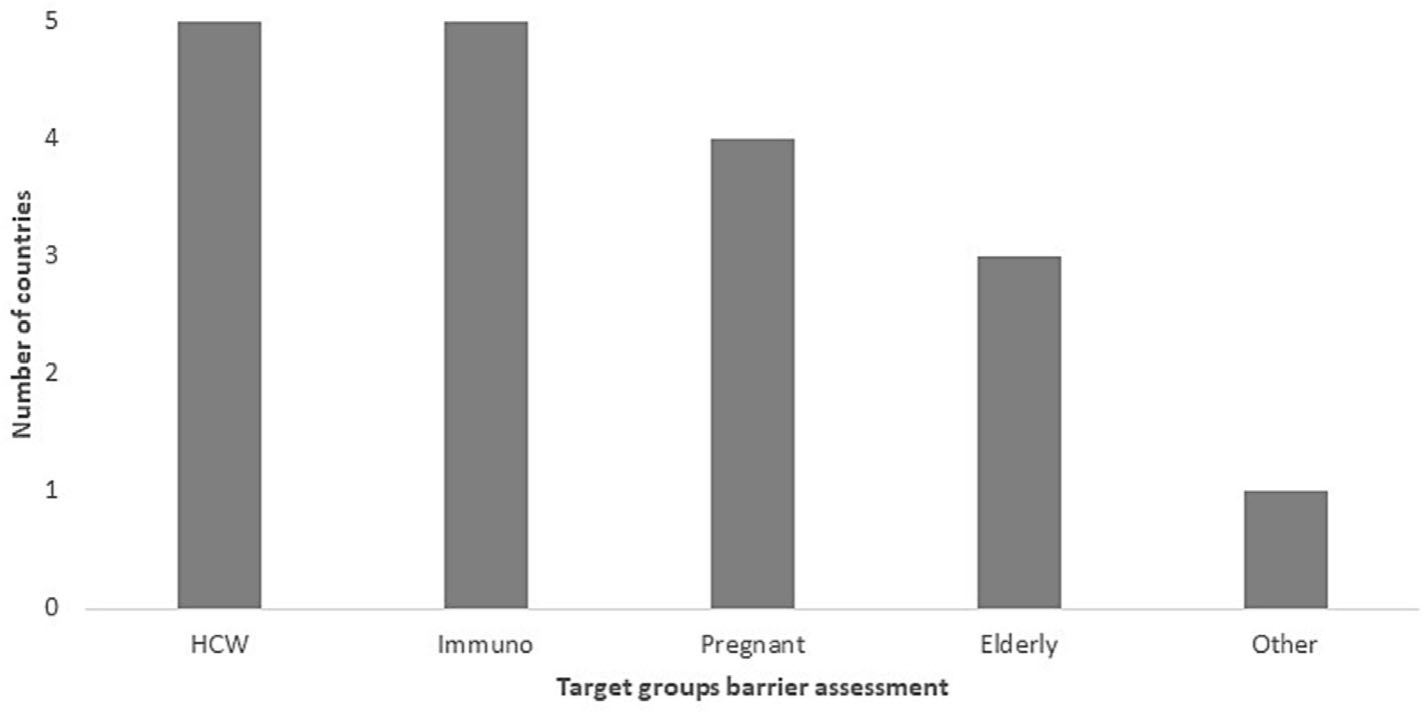
Countries of the WHO Eastern Mediterranean Region that have conducted target groups barrier assessments, 2022.

Ten (10/16; 63%) countries reported conducting routine awareness raising campaigns on influenza vaccination; the same 10 (Bahrain, Egypt, Iran, Kuwait, Morocco, Oman, Qatar, Saudi Arabia, Tunisia, and UAE) also reported that they conducted such campaigns in the most recent (2021–2022) season. Common platforms used to raise awareness were social media, posters in health facilities, and official websites (Figure 2). Only five countries (5/16; 31%) reported conducting barrier assessments with the target groups (see Figure 3). Only UAE reported conducting a barrier assessment of the general public.

#### 3.1.7 Influenza disease and vaccination information sharing

In terms of data sharing, the most commonly performed assessments were on burden of influenza, with 44% (7/16) countries conducting them in 2012, 2013, 2014, 2016, 2018, and 2022. Six of these assessments were published, but there were no economic burden assessments done by any of the countries.

#### 3.1.8 COVID‐19 vaccine deployment and influenza vaccination

The COVID‐19 pandemic has impacted all health systems globally, including influenza vaccination programs. However, 11 (69%) countries reported that COVID‐19 had an impact on influenza vaccine supply management. Majority (9/11) of countries reported increases in procurement and supply of influenza vaccine, while two countries reported decreases in procurement due to COVID‐19.

**FIGURE 4:**
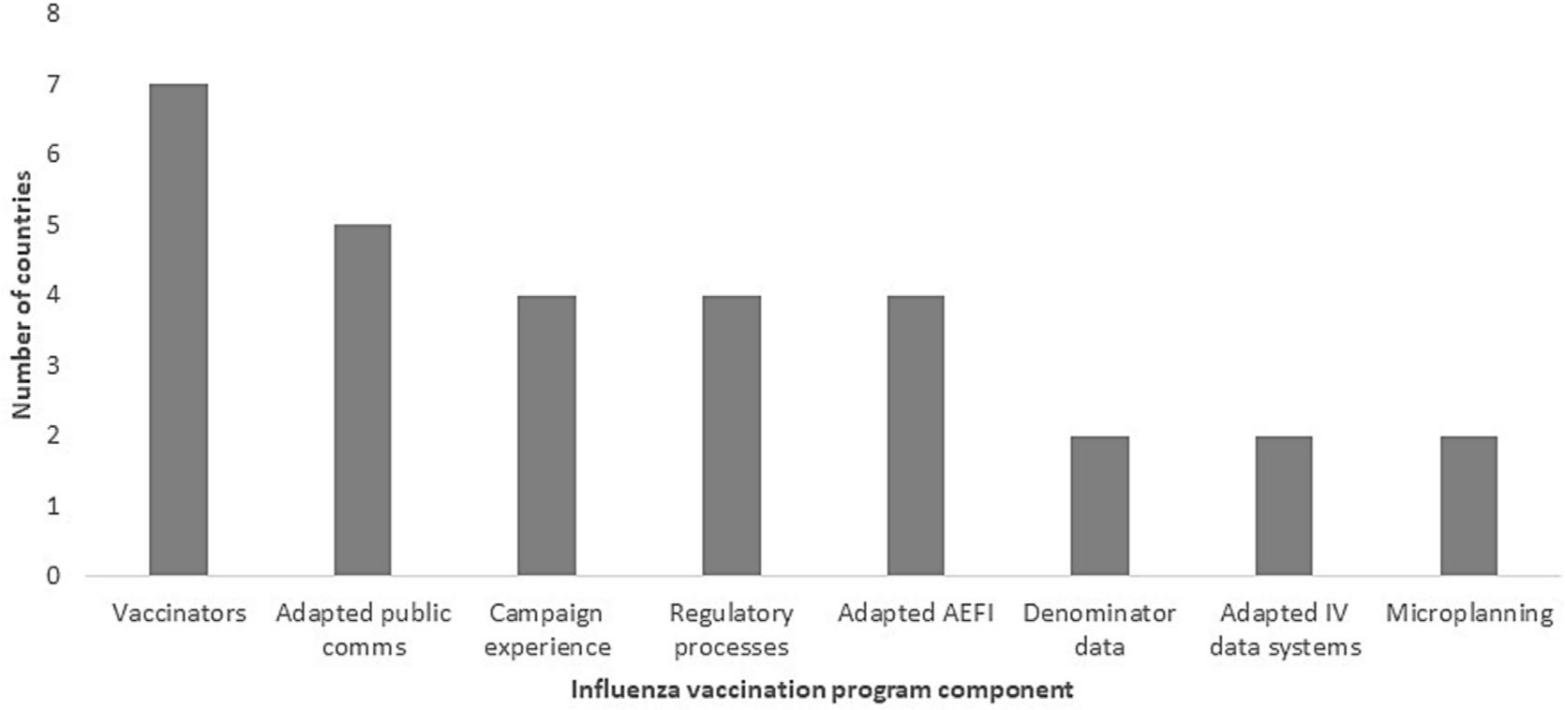
Influenza vaccine program component repurposed/used for COVID‐19 vaccine deployment of WHO Eastern Mediterranean Regional countries, 2022.

In terms of repurposing/using existing influenza vaccination program components for COVID‐19 vaccine deployment, eight (50%) countries in the region reported repurposing/using existing program components for COVID‐19 vaccine deployment (Figure 4).

### 3.2 Difference between 2016 to 2022 in the EMR

In 2022, the regional influenza vaccination survey was conducted based on the questions and variables of 2016 EMR Regional Survey. This survey was complimentary to the JRF that is conducted each year. In the 2016 survey, 20 countries responded; only exception were Bahrain and Djibouti. In our analysis, we only considered the variables that were common in both surveys (Table 2).

**TABLE 2:** Variables under each area available in both surveys (2016–2017/2021–2022).

Policy	Vaccine delivery	Vaccination coverage and data
Have policy Have recommendations National Regulatory Authority (NRA) licensure SAGE recommended target groups	Delivery outlets Vaccine cost Type of vaccine Formulation Vaccine availability Doses distributed	Data collection Vaccination coverage for target groups

#### 3.2.1 Policy

In 2016 and 2022, out of the 22 countries, 15 (68%) and 14 (64%) reported, respectively, to have a national policy. The only country that was exception in both surveys was Jordan, as it reported having no formal national policy, although they did circulate recommendations annually for influenza vaccine use in their country. Fifteen countries compared to 14 countries in 2022 survey reported using the national regulatory authorities to license influenza vaccination, with Lebanon reporting this was not the case in 2022.

**FIGURE 5:**
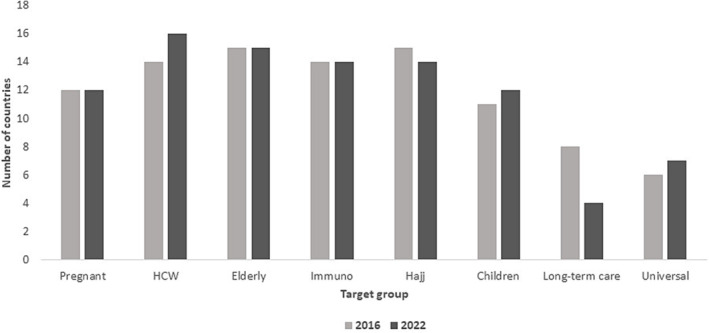
Number of WHO Eastern Mediterranean Regional countries reporting of inclusion of SAGE recommended target groups for influenza vaccination in 2016 and 2022.

Overall, the number of countries has remained stable for each target group, with the greatest discrepancy between the two time periods being the inclusion of long‐term care facilities, with a 50% drop between the two time periods. Promisingly, there have been slight increases in inclusions of HCWs, children, and universal coverage (Figure 5).

#### 3.2.2 Vaccine delivery

**FIGURE 6:**
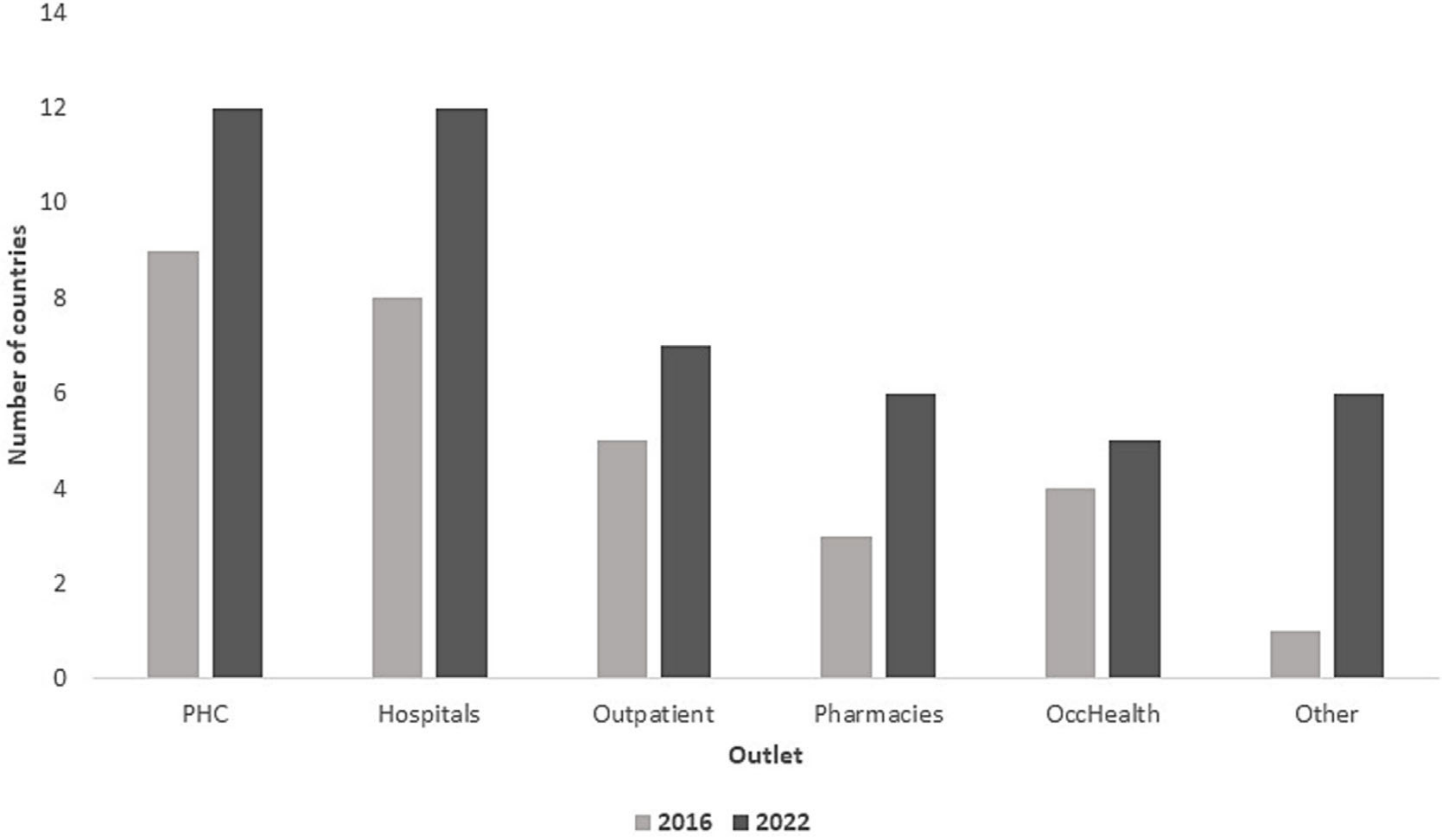
Influenza vaccination delivery outlets reported by WHO Eastern Mediterranean Regional countries in 2016 and 2022.

For both time periods, the most common outlet for vaccine delivery was primary health centers (9 in 2016 and 12 in 2022), followed closely by hospitals (Figure 6).

**FIGURE 7:**
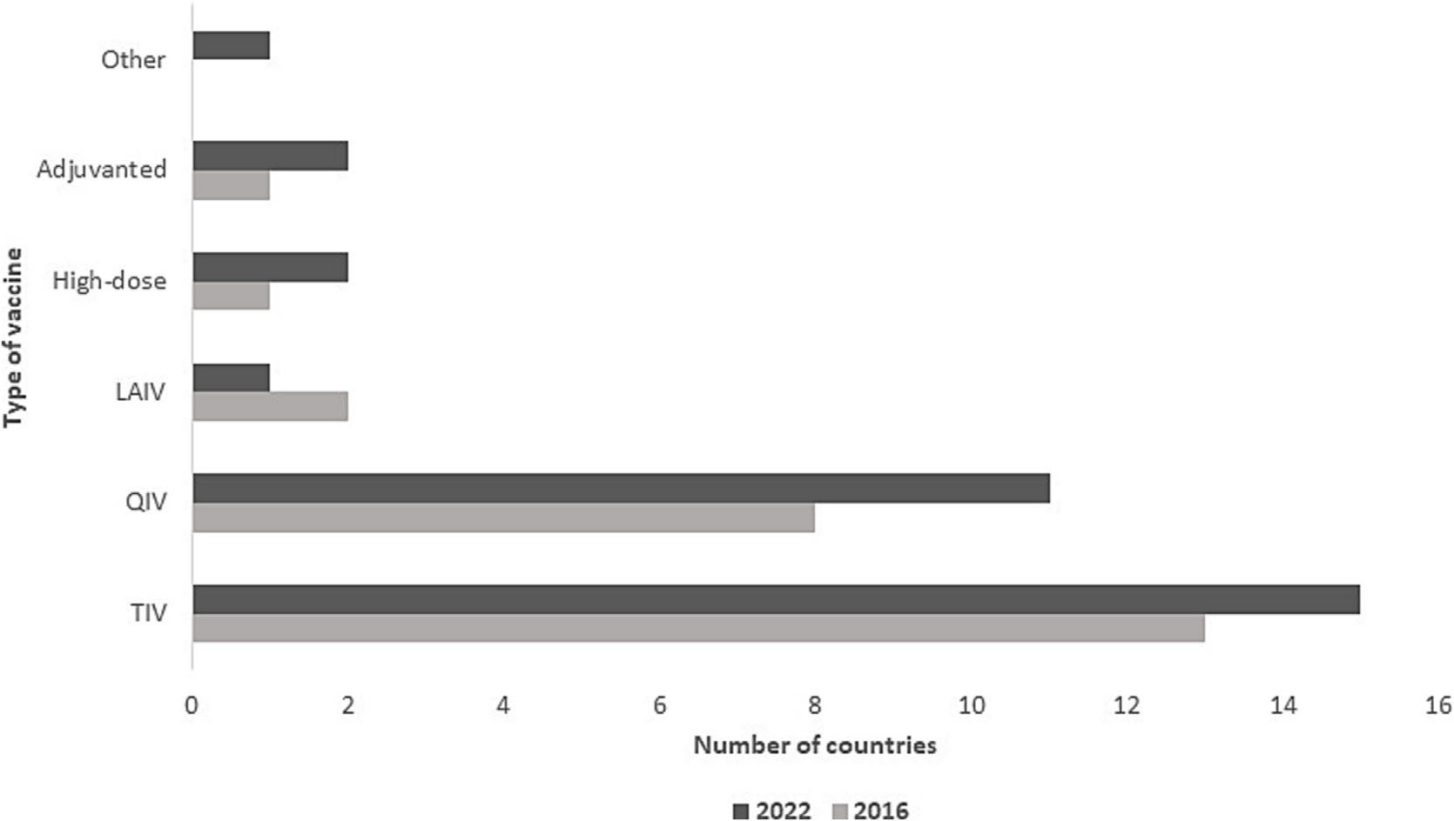
Types of influenza vaccine in use in the WHO Eastern Mediterranean Regional countries in 2016 and 2022.

Cost of influenza vaccination was difficult to report, as in 2016, there was not systematic capture of specific target groups. However, there was a small increase in terms of countries offering free influenza vaccines from 12 (2016) to 13 (2022). In the intervening time periods, there was an increase in variety of influenza vaccine given to population, but TIV was still the dominant influenza vaccine of choice. A split variant design was reported by Saudi Arabia in 2022 (Figure 7).

Influenza vaccine was generally available in 10–15 countries over the time period. However, there was no correlation between the number of countries that had vaccine available and the number of doses distributed.

#### 3.2.3 Vaccination coverage and data

Routine data collection of target groups improved from 6 countries in 2016–2017 to 14 in 2021–2022. In terms of vaccination coverage, over time, there was an increase in the number of countries reporting vaccination coverage of different target groups (Table 2).

### 3.3 Difference between high resourced countries and fragile states

To investigate the potential difference of influenza programs between high resource states and fragile states, a sub‐analysis of the data was conducted. High resource states included UAE, Qatar, Kuwait, Bahrain, Saudi Arabia, and Oman, while fragile states included Iraq, Libya, Somalia, Sudan, Yemen, Afghanistan, Syria, and Palestine.

#### 3.3.1 Policy

All high resource countries (6/6) have national policy and use national regulatory agencies to license influenza vaccines in their respective countries. In comparison, only half of the fragile states have national policies and recommendations (4/8), with only three of eight using their national regulatory agencies to license the vaccine.

**FIGURE 8:**
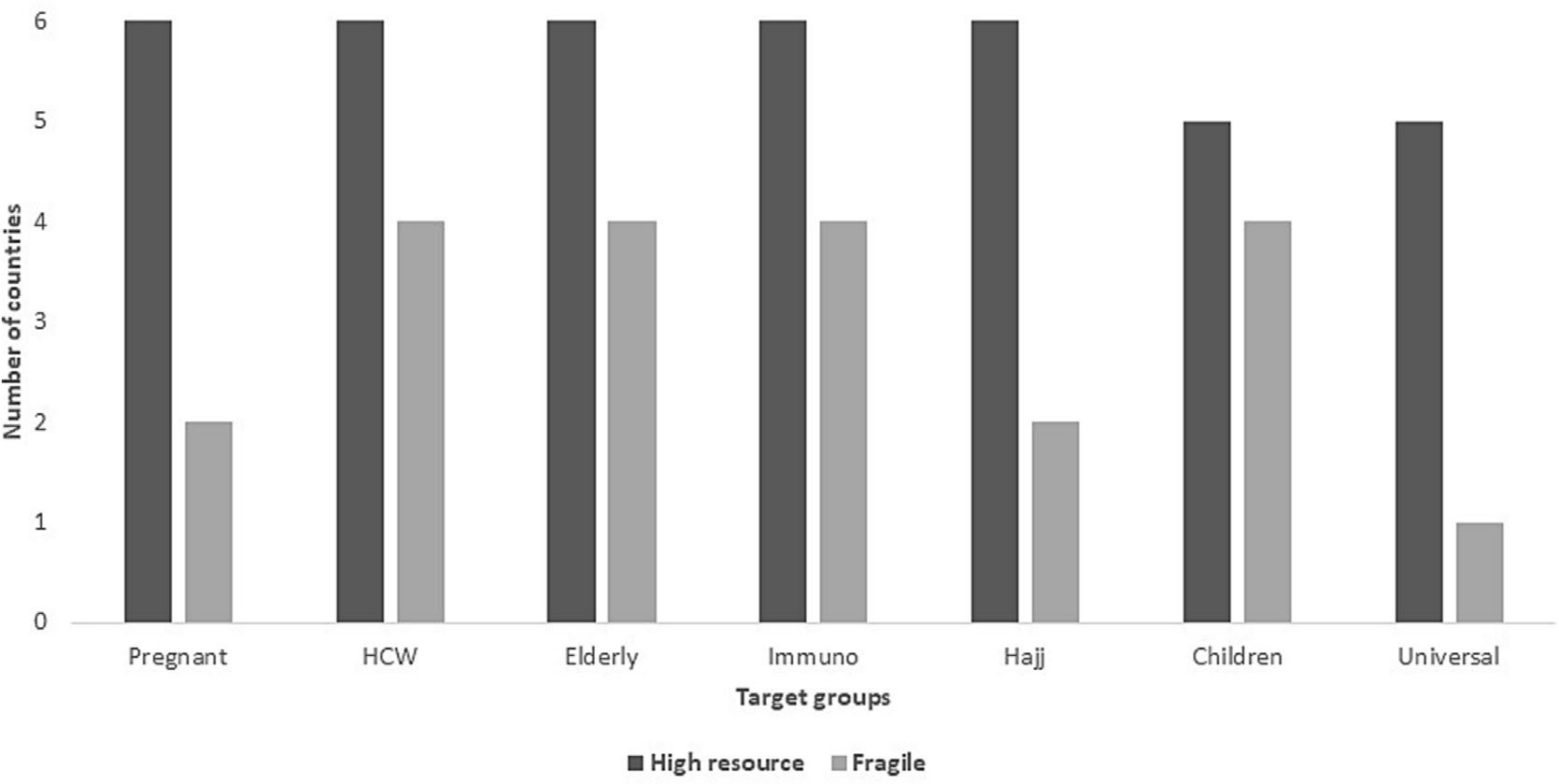
Distribution of SAGE recommended target groups included in country policies/recommendations in the WHO Eastern Mediterranean Regional countries in 2016 and 2022.

SAGE recommended target groups were almost covered by all high‐income states and five also reported having universal coverage (Figure 8).

#### 3.3.2 Vaccine delivery

In terms of vaccine cost, high resource countries provided influenza vaccine for free to all, whereas only two (25%) countries in fragile did so.

**FIGURE 9:**
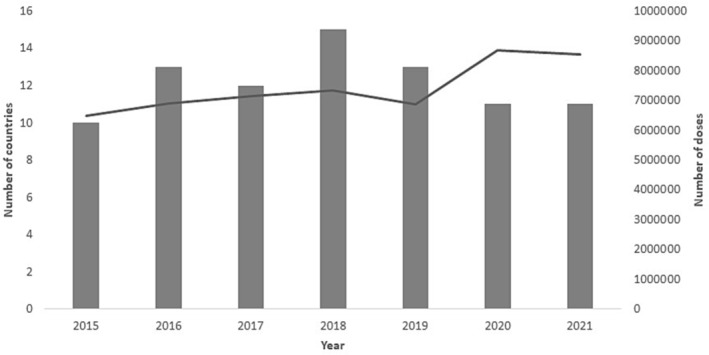
Availability of influenza vaccines and doses distributed in the WHO Eastern Mediterranean Regional countries from 2015–2021.

Consistently, high resource countries have had a higher number of doses distributed to their population as compared to fragile states. Availability of vaccine has been more stable in high resource countries, than fragile states, except for the year 2017 (Figure 9).

#### 3.3.3 Vaccination coverage and data

**FIGURE 10:**
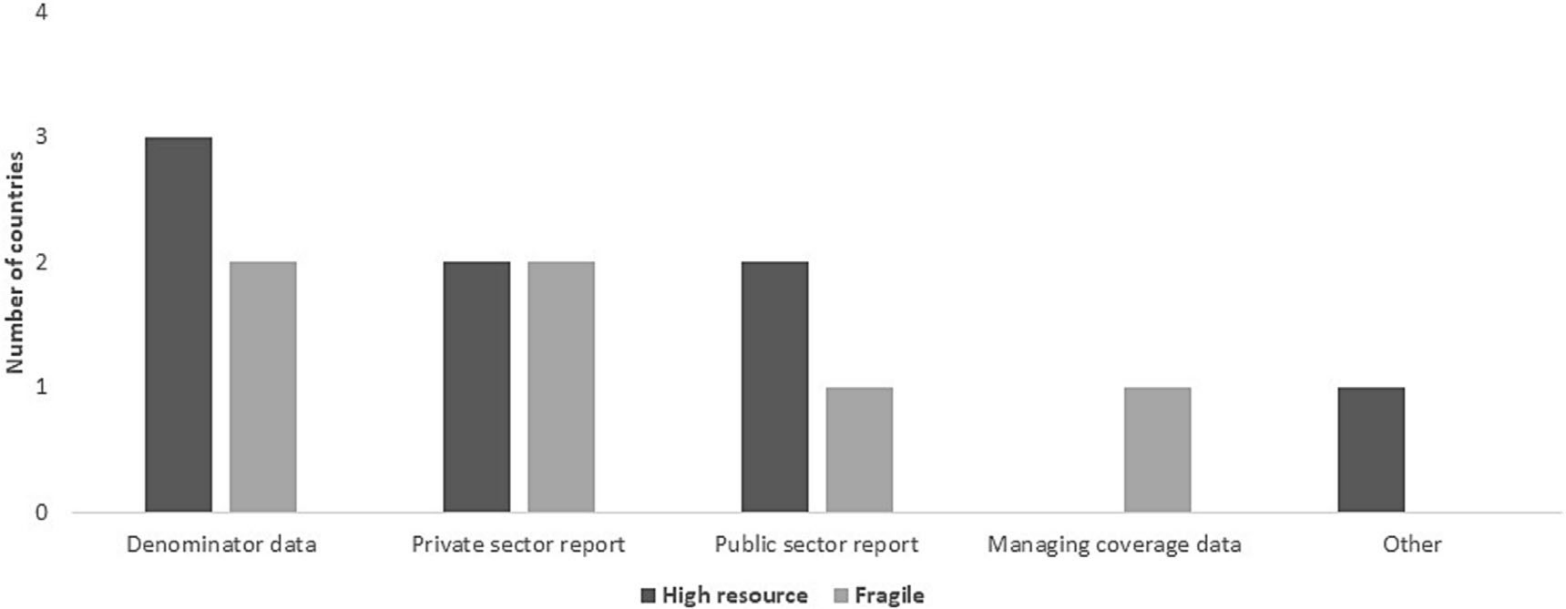
Vaccination coverage data collection challenges by the two country groups in the WHO Eastern Mediterranean Regional countries in 2016 and 2022.

Routine target group coverage collection was conducted in five (83%) high resource countries in comparison to one (17%) in the fragile states. This was also the case with having population/denominator data and collection as part of routine immunization, where five high resource countries reported four (67%) and only one (17%) fragile state reported. No fragile state reported target vaccination coverage or vaccination coverage rates for the seasons 2018–2021 (Figure 10).

#### 3.3.4 Influenza vaccination program

For high resource countries, the majority have included influenza vaccine into their respective NIPs (4/6; 67%), with Oman also planning to do this. All high resource countries have included influenza vaccine safety monitoring into their routine immunization safety monitoring. This was not the case for fragile states, with none having included influenza vaccine into their respective NIPs nor they are planning for future. In addition, all high resource countries routinely conducted awareness campaigns, including for the most recent season, in comparison to none of the fragile states.

#### 3.3.5 Influenza disease and vaccination information sharing

In terms of information sharing, no fragile states conducted or published barrier assessments, burden of influenza, or economic burden assessments. Fifty percent of high resource countries conducted barrier and influenza BoD assessments, but only two countries (Oman and Kuwait) have published their burden of disease assessments. None of the high resource countries have conducted and/or published any economic burden assessments. This is not the case for influenza hospitalizations and deaths. All high resource countries and the majority (5/6; 83%) of fragile states collect hospitalization death data.

#### 3.3.6 COVID‐19 vaccine deployment and influenza vaccination

The impact of COVID‐19 on influenza vaccine supply witnessed an increase in high resource countries (5/6; 83%) but was mixed for fragile, with Iraq reporting a decrease in supply and Palestine reporting an increase. No fragile states reported the use of their influenza vaccination program components to be repurposed for use in COVID‐19 vaccine deployment. In comparison, 67% (4/6) high resource countries reported that they repurposed parts of their influenza vaccination programs for COVID‐19 vaccine deployment.

## 4 DISCUSSION

The influenza vaccination situation in the EMR is varied, with some countries reporting having well established policies, programs, and strong supply of vaccine, whereas others having no policy or program and no supply systems; these differences may be due to resources, social, political, and economic variations.^19^ This makes a strong case for development of regional roadmap for strengthening of influenza vaccination policies and programs with a targeted approach to address the different contexts of influenza vaccine use and to strengthen the seasonal influenza vaccination programs in EMR.^20^

Since 2016, there has been a little change in the number of countries that have a formal national policy for influenza vaccination in their respective countries (64%) but with a larger proportion than was globally reported (59%).^15^ Even among those regional countries that do, there is not universal policy coverage for all SAGE recommended groups as universal health coverage is not consistent in the region due to inconsistency in political, governance, decision making, and implementation factors.^21^ In terms of the vaccine type used, TIV is the vaccine most commonly used, and there has been an increase in the more recent years of QIV use in the region that has public health and economic importance.^22^

Over the time period, there was a greater variety of outlets used to deliver the vaccine, which is good, as this will improve access, particularly the use of outreach, such as mobile units, home visits, and rehabilitation centers, as the target groups would benefit from these services. Around 81% of countries reported providing influenza vaccine free to some of their target groups as providing seasonal influenza vaccine with no cost will increase the acceptancy and the willingness to vaccine uptake.^23^ The main supported groups were HCWs, the elderly and groups with chronic diseases, which are the most common causes for receiving influenza vaccine globally.^24^ The majority of countries, reporting universally providing vaccine free of charge, are the high resourced countries and that is mainly due to income and affordability by those countries.^25^

Few countries reported wide ranges vaccination coverage over time with no clear trend of improvement, which has the same differences and inconsistency among most of countries depending on a lot of factors but especially socioeconomic status.^26^ The main issue with reporting of vaccination coverage data is primarily from lack of data on vaccine delivery via the private sector. This is problematic for countries to track their progress, improve utilization, and uptake of influenza vaccination. For obvious reasons, high resource countries reported more coverage data than fragile ones.^33^ This is because these programs have been established for many years; they have a better understanding of gaps, better documentation, data quality, and share their information by using global and regional platforms.

Awareness campaigns were reported by majority of countries with a national policy or recommendations, while the same number also reported conducting awareness raising campaigns in the most recent season, using primarily social media and health facility posters, highlighting the importance and positive effect of awareness raising campaigns.^27^ Very few countries reported conducting barrier assessments to understand the challenges of influenza vaccine uptake and utilization among their target groups for addressing seasonal influenza vaccine attitudes and misconception.^28^

COVID‐19 vaccine deployment witnessed 50% of countries repurpose/use existing program components from their influenza vaccination programs to support the roll out. It is not clear why some did not use their influenza vaccination programs to benefit from the availability of vaccine delivery system, infrastructure, and resources.^29^

## 5 CONCLUSION

WHO EMR faces several challenges regarding seasonal influenza vaccination especially during COVID‐19 pandemic. Despite the progress and efforts made over the years, to improve and enhance the effect of seasonal influenza vaccination programs among EMR countries, one third of them still lack the presence of vaccine policy. High resource countries have a national policy and use national regulatory agencies to license influenza vaccines in their countries. Further efforts in the form of trainings and joint advocacy missions are needed to improve orientation and awareness, strengthen systems, and enhance the confidence of people. Assisting and providing countries with strategic guidance, technical support, and evidence is essential to make their health systems better prepared for flu seasons. WHO should work actively with countries to develop a roadmap for influenza vaccine uptake and utilization, considering many countries would plan to increase their use of influenza vaccine and to be focused on the highest risk population to maximize the health gains from these programs. Barrier assessments, influenza burden of disease assessments, economic burden assessments, and generation of local knowledge are strongly recommended to improve vaccine acceptance and use. Monitoring and evaluation of seasonal influenza vaccination program implementation and to share the experience among EMR countries are another important step to reduce the gaps among different settings and situations.

## AUTHOR CONTRIBUTIONS

**Rania Abdelkader Mohamed Attia:** Conceptualization; data curation; formal analysis; investigation; methodology; validation; writing—original draft; writing—review and editing. **Abdinasir Abubakar:** Methodology; supervision; writing—review and editing. **Joseph Bresee:** Writing—review and editing. **Osama Mere:** Methodology; visualization; writing—review and editing. **Wasiq Khan:** Conceptualization; methodology; supervision; writing—review and editing.

## CONFLICT OF INTEREST STATEMENT

The authors report no conflict of interest.

### PEER REVIEW

The peer review history for this article is available at https://www.webofscience.com/api/gateway/wos/peer-review/10.1111/irv.13126.

## Data Availability

The data that support the findings of this study are available upon reasonable request from the corresponding author. The data are not publicly available due to privacy.
